# Soldiers’ Health

**Published:** 1861-06

**Authors:** 


### SOLDIERS' HEALTH.

*From Hall's New-York Journal of Health.*

1. In any ordinary campaign, sickness disables or destroys three times as many as the sword.

2. On a march, from April to November, the entire clothing should be a colored flannel shirt, with a loosely-buttoned collar, cotton drawers, woolen pantaloons, shoes and stockings, and a light-colored felt hat, with broad brim to protect the neck, eyes, and face from the glare of the sun and from the rain, and a substantial but not heavy coat when off duty.

3. Sun-stroke may be prevented by wearing a silk handkerchief in the hat, or a white linen hood hat-cover, extending like a cape over the neck and shoulders.

4. Colored blankets are best, and if lined with brown drilling the warmth and durability are doubled, while the protection against dampness from lying on the ground, is almost complete.

5. Never lie or sit down on the grass or bare earth for a moment; rather use your hat—a handkerchief even, is a great protection. The warmer you are, the greater need for this precaution, as a damp vapor is immediately generated, to be absorbed by the clothing, and to cool you off too rapidly.

6. While marching, or on other active duty, the more thirsty you are, the more essential is it to safety of life itself, to rinse out the mouth two or three times, and *then* take a swallow of water at a time, with short intervals. A brave French general, on a forced march, fell dead on the instant, by drinking largely of cold water, when snow was on the ground.

7. Abundant sleep is essential to bodily efficiency, and to that alertness of mind which is all-important in an engagement; and few things more certainly and more effectually prevent sound sleep than eating heartily after sun-down, especially after a heavy march or desperate battle.

8. Nothing is more certain to secure endurance and capability of long-continued effort, than the avoidance of every thing as a drink except cold water, NOT excluding coffee at breakfast. Drink even cold water very slowly.

9. After any sort of exhausting effort, a cup of coffee, hot or cold, is an admirable sustainer of the strength, until nature begins to recover herself.

10 Unless after a long abstinence or great fatigue, do not eat very heartily just before a great undertaking; because the nervous power is irresistibly drawn to the stomach to manage the food eaten, thus drawing off that supply which the brain and muscles so much need.

11. If persons will drink brandy, it is incomparably safer to do so *after* an effort than before; for it can give only a transient strength, lasting but a few minutes; but as it can never be known how long any given effort is to be kept in continuance, and if longer than the few minutes, the body becomes more feeble than it would have been without the stimulus, it is clear that its use *before* an effort is always hazardous, and is always unwise.

12. Never go to sleep, especially after a great effort, even in hot weather, without some covering over you.

13. Under all circumstances, rather than lie down on the bare ground, lie in the hollow of two logs placed together, or across several smaller pieces of wood, laid side by side; or sit on your hat, leaning against a tree. A nap of ten or fifteen minutes in that position will refresh you more than an hour on the bare earth, with the additional advantage of perfect safety.

14. A *cut* is less dangerous than a bullet-wound, and heals more rapidly.

15. If from any wound the blood spirts out in jets, instead of a steady stream, you will die in a few minutes unless it is remedied; because an artery has been divided, and that takes the blood direct from the fountain of life. To stop this instantly, tie a handkerchief or other cloth very loosely BETWEEN! ! the wound and the heart; put a stick, bayonet, or ramrod *between* the skin and the handkerchief, and twist it around until the bleeding ceases, and keep it thus until the surgeon arrives.

16. If the blood flows in a slow, regular stream, a vein has been pierced, and the handkerchief must be on the other side of the wound from the heart; that is, *below* the wound.

17. A bullet through the abdomen (belly or stomach) is more certainly fatal than if aimed at the head or heart; for in the latter cases the ball is often glanced off by the bone, or follows round it under the skin; but when it enters the stomach or bowels, from any direction, death is inevitable under almost all circumstances, but is scarcely ever instantaneous. Generally the person lives a day or two with perfect clearness of intellect, often *not* suffering greatly. The practical bearing of this statement in reference to the great future is clear.

18. Let the whole beard grow, but not longer than some three inches. This strengthens and thickens its growth, and thus makes a more perfect protection for the lungs against dust, and of the throat against winds and cold in winter, while in the summer a greater perspiration of the skin is induced, with an increase of evaporation; hence, greater coolness of the parts on the outside, while the throat is less feverish, thirsty, and dry.

19. Avoid fats and fat meats in summer and in all warm days.

20. Whenever possible, take a plunge into any lake or running stream every morning, as soon as you get up; if none at hand, endeavor to wash the body all over as soon as you leave your bed, for personal cleanliness acts like a charm against all diseases, always either warding them off altogether, or greatly mitigating their severity and shortening their duration.

21. Keep the hair of the head closely cut, say within an inch and a half of the scalp in every part, repeated on the first of each month, and wash the whole scalp plentifully in cold water every morning.

22. Wear woolen stockings and easy-fitting shoes, keeping the toe and finger-nails always cut moderately close.

23. It is more important to wash the feet well every night, than to wash the face and hands of mornings; because it aids to keep the skin and nails soft, and to prevent chafings, blisters, and corns, all of which greatly interfere with a soldier's duty.

24. The most universally safe position, after all stunnings, hurts, and wounds, is that of being placed on the back, the head being elevated three or four inches only; aiding more than any one thing else can do, to equalize and restore the proper circulation of the blood.

25. The more weary you are after a march or other work, the more easily will you take cold, if you remain still after it is over, unless, the moment you cease motion, you throw a coat or blanket over your shoulders. This precaution should be taken in the warmest weather, especially if there is even a slight air stirring.

26. The greatest physical kindness you can show a severely-wounded comrade is first to place him on his back, and then run with all your might for some water to drink; not a second ought to be lost. If no vessel is at hand, take your hat; if no hat, off with your shirt, wring it out once, tie the arms in a knot, as also the lower end, thus making a bag, open at the neck only. A fleet person can convey a bucketful half a mile in this way. I've seen a dying man clutch at a single drop of water from the fingers' end, with the voraciousness of a famished tiger.

27. If wet to the skin by rain or by swimming rivers, keep in motion until the clothes are dried, and no harm will result.

28. Whenever it is possible, do, by all means, when you have to use water for cooking or drinking from ponds or sluggish streams, boil it well, and when cool, shake it, or stir it, so that the oxygen of the air shall get to it, which greatly improves it for drinking. This boiling arrests the process of fermentation which arises from the presence of organic and inorganic impurities, thus tending to prevent cholera and all bowel diseases. If there is no time for boiling, at least strain it through a cloth, even if you have to use a shirt or trowser-leg.

29. Twelve men are hit in battle, dressed in red, where there are only five, dressed in a bluish grey, a difference of more than two to one; green, seven; brown, six.

30. Water can be made almost ice-cool in the hottest weather, by closely enveloping a filled canteen, or other vessel, with woolen cloth kept plentifully wetted and exposed.

31. While on a march, lie down the moment you halt for a rest; every minute spent in that position refreshes more than five minutes standing or loitering about.

32. A daily evacuation of the bowels is indispensable to bodily health, vigor, and endurance; this is promoted in many cases, by stirring a table-spoonful of corn (Indian) meal in a glass of water, and drinking it on rising in the morning.

33. Loose Bowels, namely, acting more than once a day, with a feeling of debility afterward, is the first step toward cholera; the best remedy is instant and perfect quietude of body, eating nothing but boiled rice with or without boiled milk; in more decided cases, a woolen flannel, with two thicknesses in front, should be bound tightly around the abdomen, especially if marching is a necessity.

34. To have "been to the wars," is a life-long honor, increasing with advancing years, while to have died in defense of your country will be the boast and the glory of your children's children.

### SOLDIERS' HEALTH.

*From Hall's New-York Journal of Health.*

1. IN any ordinary campaign, sickness disables or destroys three times as many as the sword.

2. On a march, from April to November, the entire clothing should be a colored flannel shirt, with a loosely-buttoned collar, cotton drawers, woolen pantaloons, shoes and stockings, and a light-colored felt hat, with broad brim to protect the neck, eyes, and face from the glare of the sun and from the rain, and a substantial but not heavy coat when off duty.

3. SUN-STROKE may be prevented by wearing a silk handkerchief in the hat, or a white linen hood hat-cover, extending like a cape over the neck and shoulders.

4. COLORED blankets are best, and if lined with brown drilling the warmth and durability are doubled, while the protection against dampness from lying on the ground, is almost complete.

5. Never lie or sit down on the grass or bare earth for a moment; rather use your hat—a handkerchief even, is a great protection. The warmer you are, the greater need for this precaution, as a damp vapor is immediately generated, to be absorbed by the clothing, and to cool you off too rapidly.

6. While marching, or on other active duty, the more thirsty you are, the more essential is it to safety of life itself, to rinse out the mouth two or three times, and *then* take a swallow of water at a time, with short intervals. A brave French general, on a forced march, fell dead on the instant, by drinking largely of cold water, when snow was on the ground.

7. Abundant sleep is essential to bodily efficiency, and to that alertness of mind which is all-important in an engagement; and few things more certainly and more effectually prevent sound sleep than eating heartily after sun-down, especially after a heavy march or desperate battle.

8. Nothing is more certain to secure endurance and capability of long-continued effort, than the avoidance of every thing as a drink except cold water, NOT excluding coffee at breakfast. Drink even cold water very slowly.

9. After any sort of exhausting effort, a cup of coffee, hot or cold, is an admirable sustainer of the strength, until nature begins to recover herself.

10. Unless after a long abstinence or great fatigue, do not eat very heartily just before a great undertaking; because the nervous power is irresistibly drawn to the stomach to manage the food eaten, thus drawing off that supply which the brain and muscles so much need.

11. If persons will drink brandy, it is incomparably safer to do so *after* an effort than before; for it can give only a transient strength, lasting but a few minutes; but as it can never be known how long any given effort is to be kept in continuance, and if longer than the few minutes, the body becomes more feeble than it would have been without the stimulus, it is clear that its use *before* an effort is always hazardous, and is always unwise.

12. Never go to sleep, especially after a great effort, even in hot weather, without some covering over you.

13. Under all circumstances, rather than lie down on the bare ground, lie in the hollow of two logs placed together, or across several smaller pieces of wood, laid side by side; or sit on your hat, leaning against a tree. A nap of ten or fifteen minutes in that position will refresh you more than an hour on the bare earth, with the additional advantage of perfect safety.

14. A *cut* is less dangerous than a bullet-wound, and heals more rapidly.

15. If from any wound the blood spirts out in jets, instead of a steady stream, you will die in a few minutes unless it is remedied; because an artery has been divided, and that takes the blood direct from the fountain of life. To stop this instantly, tie a handkerchief or other cloth very loosely BETWEEN ! ! the wound and the heart; put a stick, bayonet, or ramrod *between* the skin and the handkerchief, and twist it around until the bleeding ceases, and keep it thus until the surgeon arrives.

16. If the blood flows in a slow, regular stream, a vein has been pierced, and the handkerchief must be on the other side of the wound from the heart; that is, *below* the wound.

17. A bullet through the abdomen (belly or stomach) is more certainly fatal than if aimed at the head or heart; for in the latter cases the ball is often glanced off by the bone, or follows round it under the skin; but when it enters the stomach or bowels, from any direction, death is inevitable under almost all circumstances, but is scarcely ever instantaneous. Generally the person lives a day or two with perfect clearness of intellect, often *not* suffering greatly. The practical bearing of this statement in reference to the great future is clear.

18. Let the whole beard grow, but not longer than some three inches. This strengthens and thickens its growth, and thus makes a more perfect protection for the lungs against dust, and of the throat against winds and cold in winter, while in the summer a greater perspiration of the skin is induced, with an increase of evaporation; hence, greater coolness of the parts on the outside, while the throat is less feverish, thirsty, and dry.

19. Avoid fats and fat meats in summer and in all warm days.

20. Whenever possible, take a plunge into any lake or running stream every morning, as soon as you get up; if none at hand, endeavor to wash the body all over as soon as you leave your bed, for personal cleanliness acts like a charm against all diseases, always either warding them off altogether, or greatly mitigating their severity and shortening their duration.

21. Keep the hair of the head closely cut, say within an inch and a half of the scalp in every part, repeated on the first of each month, and wash the whole scalp plentifully in cold water every morning.

22. Wear woolen stockings and easy-fitting shoes, keeping the toe and finger-nails always cut moderately close.

23. It is more important to wash the feet well every night, than to wash the face and hands of mornings; because it aids to keep the skin and nails soft, and to prevent chafings, blisters, and corns, all of which greatly interfere with a soldier's duty.

24. The most universally safe position, after all stunnings, hurts, and wounds, is that of being placed on the back, the head being elevated three or four inches only; aiding more than any one thing else can do, to equalize and restore the proper circulation of the blood.

25. The more weary you are after a march or other work, the more easily will you take cold, if you remain still after it is over, unless, the moment you cease motion, you throw a coat or blanket over your shoulders. This precaution should be taken in the warmest weather, especially if there is even a slight air stirring.

26. The greatest physical kindness you can show a severely-wounded comrade is first to place him on his back, and then run with all your might for some water to drink; not a second ought to be lost. If no vessel is at hand, take your hat; if no hat, off with your shirt, wring it out once, tie the arms in a knot, as also the lower end, thus making a bag, open at the neck only. A fleet person can convey a bucketful half a mile in this way. I've seen a dying man clutch at a single drop of water from the fingers' end, with the voraciousness of a famished tiger.

27. If wet to the skin by rain or by swimming rivers, keep in motion until the clothes are dried, and no harm will result.

28. Whenever it is possible, do, by all means, when you have to use water for cooking or drinking from ponds or sluggish streams, boil it well, and when cool, shake it, or stir it, so that the oxygen of the air shall get to it, which greatly improves it for drinking. This boiling arrests the process of fermentation which arises from the presence of organic and inorganic impurities, thus tending to prevent cholera and all bowel diseases. If there is no time for boiling, at least strain it through a cloth, even if you have to use a shirt or trowser-leg.

29. Twelve men are hit in battle, dressed in red, where there are only five, dressed in a bluish grey, a difference of more than two to one; green, seven; brown, six.

30. Water can be made almost ice cool in the hottest weather, by closely enveloping a filled canteen, or other vessel, with woolen cloth kept plentifully wetted and exposed.

31. While on a march, lie down the moment you halt for a rest; every minute spent in that position refreshes more than five minutes standing or loitering about.

32. A daily evacuation of the bowels is indispensable to bodily health, vigor, and endurance; this is promoted in many cases, by stirring a table-spoonful of corn (Indian) meal in a glass of water, and drinking it on rising in the morning.

33. Loose Bowels, namely, acting more than once a day, with a feeling of debility afterward, is the first step toward cholera; the best remedy is instant and perfect quietude of body, eating nothing but boiled rice with or without boiled milk; in more decided cases, a woolen flannel, with two thicknesses in front, should be bound tightly around the abdomen, especially if marching is a necessity.

34. To have "been to the wars," is a life-long honor, increasing with advancing years, while to have died in defense of your country will be the boast and the glory of your children's children.

